# Chronic parasitization by *Nosema* microsporidia causes global expression changes in core nutritional, metabolic and behavioral pathways in honey bee workers (*Apis mellifera*)

**DOI:** 10.1186/1471-2164-14-799

**Published:** 2013-11-18

**Authors:** Holly L Holt, Katherine A Aronstein, Christina M Grozinger

**Affiliations:** Department of Entomology, Center for Pollinator Research, Center for Chemical Ecology, Huck Institutes of the Life Sciences, Pennsylvania State University, University Park, Kragujevac, USA; Honey Bee Breeding, Genetics & Physiology Lab, USDA, Baton Rouge, USA; 3A Chemical Ecology Laboratory, Pennsylvania State University, Orchard Road, University Park, Kragujevac, PA 16802 USA

**Keywords:** *Nosema*, Honey bee, Behavioral maturation, Insulin signaling pathway, Innate immunity, Chronic parasite, Nutrition, Genomics, Stress

## Abstract

**Background:**

Chronic infections can profoundly affect the physiology, behavior, fitness and longevity of individuals, and may alter the organization and demography of social groups. *Nosema apis* and *Nosema ceranae* are two microsporidian parasites which chronically infect the digestive tract of honey bees (*Apis mellifera*). These parasites, in addition to other stressors, have been linked to increased mortality of individual workers and colony losses in this key pollinator species. Physiologically, *Nosema* infection damages midgut tissue, is energetically expensive and alters expression of immune genes in worker honey bees. Infection also accelerates worker transition from nursing to foraging behavior (termed behavioral maturation). Here, using microarrays, we characterized global gene expression patterns in adult worker honey bee midgut and fat body tissue in response to *Nosema* infection.

**Results:**

Our results indicate that *N. apis* infection in young workers (1 and 2 days old) disrupts midgut development. At 2 and 7 days post-infection in the fat body tissue, *N. apis* drives metabolic changes consistent with energetic costs of infection. A final experiment characterizing gene expression in the fat bodies of 14 day old workers parasitized with *N. apis* and *N. ceranae* demonstrated that *Nosema* co-infection specifically alters conserved nutritional, metabolic and hormonal pathways, including the insulin signaling pathway, which is also linked to behavioral maturation in workers. Interestingly, in all experiments, *Nosema* infection did not appear to significantly regulate overall expression of canonical immune response genes, but infection did alter expression of acute immune response genes identified in a previous study. Comparative analyses suggest that changes in nutritional/metabolic processes precede changes in behavioral maturation and immune processes.

**Conclusions:**

These genome-wide studies of expression patterns can help us disentangle the direct and indirect effects of chronic infection, and understand the molecular pathways that regulate disease symptoms.

**Electronic supplementary material:**

The online version of this article (doi:10.1186/1471-2164-14-799) contains supplementary material, which is available to authorized users.

## Background

Chronic parasitization can have profound effects on host physiology, behavior and fitness [1]. Studying the molecular mechanisms mediating host responses to prolonged parasitism can be challenging, since chronic infections can lead to complex and extensive downstream effects. Thus, the direct and indirect effects of parasites, and how these contribute to symptoms of infection, can be difficult to disentangle. In social systems, the interactions between parasites and host defense strategies may lead to changes in host social interactions and organization, which in turn can alter transmission patterns of the parasite and have effects on the viability of the social group [2]. The microsporidian parasites *Nosema apis* and *Nosema ceranae* and their honey bee (*Apis mellifera*) hosts [3] provide an excellent model for studying the molecular basis of direct and indirect effects of chronic parasitization on individuals, social interactions and social group organization.

Microsporidia are a specialized group of derived, pathogenic fungi that cause chronic, intracellular infections, generally in animal hosts [4]. In honey bees, environmental spores of *Nosema* spp. are spread via fecal/oral routes. Once ingested, spores germinate, infecting host midgut cells, and parasites replicate intracellularly as vegetative states [5]. Ultimately, new spores are produced which may infect other host cells or are voided [3]. Crowded hive environments can facilitate rapid pathogen transmission which may be counteracted by individuals’ immune systems, social immune defenses and the genetic diversity within a colony, fostered by the polyandrous behavior of queens [6]. Despite individual and social defenses, infection with either species of *Nosema* may undermine colony health. However, there is global variation in reports of species virulence in cage trials and field studies. Cage studies comparing species virulence offer mixed support for greater *N. ceranae* virulence contrasted with *N. apis*[7–10] while *N. ceranae* in particular has been regionally correlated with colony morbidity and mortality (see [3, 11] for a review and [12]). A number of factors, including experimental conditions, the presence of other stressors (e.g. pesticides, other diseases), temperature/climate and potentially *Nosema* control agents [13–19], are thought to modulate virulence and/or distribution of either parasite species, which may partially explain global patterns in *Nosema* spp. prevalence and variation in experimental outcomes. Other species-specific differences in parasite pathology, such as differences in replication rate and damage done to midgut tissue, in addition to molecular evidence (which remains to be verified via microscopy approaches) for differences in host tissue distribution, may also contribute to *N. ceranae*’s alleged greater virulence [8, 14, 15, 20, 21]. By studying gene expression patterns in tissues that are directly and systemically affected by *Nosema* species parasitization, we can begin to unravel the causative pathways that underlie host morbidity and mortality.

At the individual level, *Nosema* infection is energetically costly which may, in part, drive reported physiological and behavioral symptoms of infection in workers. Diverse studies conducted in cages and/or the field have documented infection costs. Because microsporidia are specialized pathogens with reduced metabolic capacities, they rely heavily on their hosts to furnish energy for parasite growth and reproduction [4] as indicated by the recently published Spartan genome of *N. ceranae*[22]. Indeed, *N. ceranae* infected workers are energetically impoverished: they are hungrier and more susceptible to starvation than controls [23, 24], have diminished hemolymph trehalose concentrations [25] and are less likely to feed nestmates via trophallaxis [26]. *N. apis* similarly disrupts worker nutrition and energy balance. Infected workers have altered titers of hemolymph amino acids [27]. However, one study directly comparing energetic costs across parasite species suggests that *N. apis* infection is not as energetically draining as *N. ceranae* infection [24]. More studies directly comparing energetic costs of each parasite, with longer time-frames to accommodate the chronic nature of infection are needed. Comparative functional analysis of parasite genomes may also help to explain species-specific differences in energetic costs.

Symptoms of energetic distress in infected bees are not surprising given that both species of *Nosema* destructively replicate within honey bee midgut tissue [8, 20, 28, 29] which likely impairs their hosts’ ability to digest food and/or absorb nutrients. Within midgut cells, *N. apis* and *N. ceranae* associate with host cell mitochondria [8, 30] (consistent with a general microsproidian strategy for obtaining host energy [4]) and RNAi studies indicate that *N. ceranae* commandeers host ATP through ATP/ADP transporters [31], further burdening host metabolism. Other physiological symptoms of infection include altered immune function. One study, by monitoring expression of select immune genes, has indicated that *N. ceranae* but not *N. apis* may immunosuppress workers [32] at 7 days post-infection. However, immune gene expression patterns in response to *N. ceranae* infection are modulated by initial spore dosage and disease incubation period [33].

Infection with either species of *Nosema* alters worker behavior by accelerating worker transition from nursing to foraging activities, a process termed behavioral maturation [34–38]. The molecular and physiological mechanisms by which *Nosema* infection accelerates worker behavioral maturation have not been fully characterized. Workers begin their adult lives with the in-hive task of brood care, shifting to other duties such as queen attendance or nest guarding and finally to foraging as they age [39]. The transition from nursing to foraging behavior is driven/accompanied by large-scale, internal changes in worker nutrition, metabolism, hormonal profile and physiology [39, 40] in addition to external signals from the colony, including pheromones released by the brood, workers and queen [41]. Compared to nurse bees, foragers have lower lipid stores in their abdominal fat body tissue [42], decreased expression of genes related to lipid and protein metabolism, and increased expression of genes related carbohydrate metabolism [43]. Consequently, inhibition of fat accumulation can accelerate worker transition to a forager state [44] and poor diet in young adult workers changes expression of some genes to a forager-like profile [43]. Foragers also have higher levels of juvenile hormone (JH) and lower levels of the storage protein vitellogenin (Vg), which negatively interacts with JH to mediate behavioral maturation [45]. Artificially increasing JH titers [46] or decreasing Vg levels can accelerate the transition to foraging [47, 48]. Importantly, the JH/Vg regulatory pathway interacts with worker nutritional status and the insulin signaling pathway, which has also been linked to behavioral maturation [49–51].

*Nosema* may accelerate behavioral maturation in workers by modifying host nutritional, metabolic and hormonal attributes. *Nosema* broadly and negatively impacts worker energetic status which may drive changes in behavioral maturation [23–25]. Studies have also found mixed effects of *N. ceranae* on worker expression levels of vitellogenin (*vg*) and circulating JH titers. While one study has reported negligible impact of *N. ceranae* on caged worker *vg* expression at several times post-infection [33], others have found that infection reduces *vg* expression at 7 days post-infection [32] (which is consistent with precocious foraging) or causes aberrant patterns of *vg* expression in caged workers, but no difference in expression between workers housed in colonies [36]. Also, JH titers are elevated in workers infected with *N. ceranae* and *N. apis* (consistent with earlier foraging), however, the degree to which JH titers are raised varies with *Nosema* species and with parasite strain [36, 52].

Because worker nutritional, metabolic and hormonal pathways interact [40], it is unclear if: (1) *Nosema* precipitates behavioral maturation by modulating expression of genes that regulate behavioral maturation, and/or (2) *Nosema* imposes such stringent metabolic costs that host nutritional status is diminished, driving changes in metabolic or hormonal processes that regulate maturation, and/or (3) *Nosema* infection interferes with nutrient uptake, resulting in cascading effects on metabolism and behavioral maturation. In addition, because a large number of physiological changes normally accompany behavioral maturation, it is difficult to determine whether other symptoms of *Nosema* infection arise from pathological changes mediated directly by infection versus changes mediated by accelerated development. For example, changes in worker immune function are linked to worker behavioral state which correlates with age [53, 54].

Here, we used whole genome microarrays to monitor global gene expression patterns in honey bee worker midgut and fat body tissue and to identify molecular processes associated with the symptoms of *Nosema* infection. We characterized gene expression in midgut tissue infected with *N. apis* at 1 and 2 days post-infection (pi) and infected fat body tissue at 2 and 7 days pi. We also examined gene expression in co-infected (*N. apis* and *N. ceranae*) fat body tissue at 14 days pi. We sought to capture early, local immune responses in midgut tissue, the initial site of infection. We also investigated immune, hormonal and metabolic changes in fat body tissue which is an important regulator of insect nutrient stores, development and systemic immunity [55, 56]. We compared our lists of genes significantly regulated by infection to: a list of genes significantly regulated by *N. ceranae* infection in worker midgut tissue [28], genes associated with canonical immune responses [57], genes associated with acute responses to general immune challenges [58], genes associated with worker behavioral maturation, and genes associated with robust or restrictive diets [43]. Though these studies do not allow us to separate species-specific effects of *Nosema* infection, the results from these experiments provide a holistic view of how *Nosema* impacts gene expression in these worker tissues. Taken together, these findings allow us to better understand the molecular mechanisms regulating host-parasite interactions in this system, and begin to evaluate interactions between *Nosema* infection and host nutrition, physiology and behavioral maturation.

## Results

### Effects of *Nosema apis* infection on midgut and fat body gene expression

PCR of midgut tissue confirmed that samples collected in 2008 (midguts collected from bees 1, 2 and 7-days post-infection) had *N. apis* infections only (Additional file 1: Figure S1A). We used microarrays to characterize global gene expression in the midguts collected 1 and 2 days post-infection (pi) and fat bodies collected 2 and 7 days pi. After removing transcripts with expression levels below background, 11,746 and 12,492 transcripts for midgut and fat body tissues were retained for further analysis. Transcripts with significant expression differences (incorporating transcripts with significant age, treatment and age x treatment effects) for each tissue were determined using a multivariate ANOVA. We identified 736 and 2,343 unique significantly, differentially expressed transcripts in midgut and fat body tissues respectively (FDR < 0.001, Additional file 2: Table S1 and Additional file 2: Table S2).

**Figure 1:**
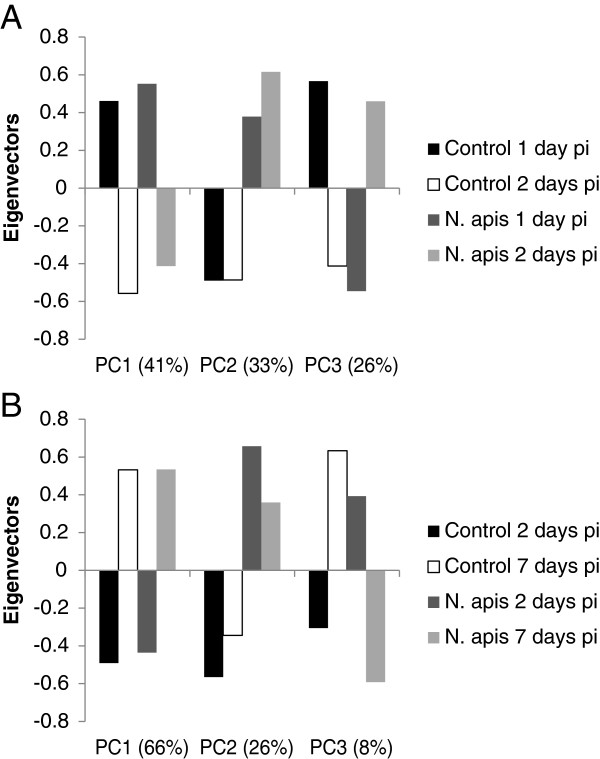
**Principal components analysis. A)** Analysis of expression patterns in midgut tissue at 1 and 2 days pi with *N. apis*. **B)** Analysis of expression patterns in fat body tissue at 2 and 7 days pi with *N. apis*. For each analysis, three PCs were identified, corresponding to age, infection status, and an age x infection interaction. The percentage of variation in transcript expression patterns explained by each PC is shown in the x-axis labels.

Principal components analysis (Figure 1) revealed the effects of treatment and age on gene expression patterns in the two tissues. For the midguts: (1) age explained 41% of the variability, (2) treatment explained 33% of the variability and, (3) an age x treatment interaction explained 26% of the variability (Figure 1A). For the fat bodies: (1) age explained 66% of the variability (2) treatment explained 26% of the variability and (3) an age x treatment interaction explained the remaining 8% of the variability (Figure 1B).

For subsequent analyses, we selected the subset of transcripts that were regulated by *N. apis* infection only (not by age or an age x treatment interaction). We found that 150 and 278 transcripts were regulated by treatment only in the midgut and fat body tissues (Additional file 2: Table S1 and Additional file 2: Table S2). Gene ontology (GO) analysis of the 150 midgut transcripts (corresponding to 84 unique *Drosophila* orthologs) yielded over-represented clusters of genes involved in tube morphogenesis, regulation of neurogenesis, sensory perception of chemical stimuli and multicellular organismal processes (*p*,0.05, Table 1). Analysis of the 278 fat body transcripts (corresponding to 201 unique *Drosophila* orthologs) yielded over-represented clusters of genes involved in metabolic processes, mitochondrial membrane organization, lipid metabolic processes, gene expression, ncRNA processing, and regulation of apoptosis (*p* < 0.05, Table 1).

**Table 1:** **Functional analysis of transcripts regulated by** ***N. apis*** **infection**

Tissue and timepoint (days pi)	GO term	GO biological process	***p***-value
**Midgut (1, 2)**	GO:0035239	Tube morphogenesis	9.19E-03
	GO:0050767	Regulation of neurogenesis	3.58E-02
	GO:0007606	Sensory perception of chemical stimulus	3.42E-02
	GO:0032501	Multicellular organismal process	4.99E-02
**Fat body (2, 7)**	GO:0044238	Primary metabolic process	3.65E-03
	GO:0007006	Mitochondrial membrane organization	1.02E-02
	GO:0006629	Lipid metabolic process	2.59E-02
	GO:0010467	Gene expression	2.62E-02
	GO:0034470	ncRNA processing	2.70E-02
	GO:0042981	Regulation of apoptosis	3.44E-02

Transcripts significantly regulated by infection in worker midgut and fat body tissue were selected for functional analysis and overrepresented GO processes were identified.

We also examined directional expression of the transcripts from significant GO categories. In midgut tissue, *Nosema apis* infection increased expression of transcripts involved in regulation of neurogenesis, tube morphogenesis, and multicellular organismal processes. The opposite pattern was observed for sensory perception of chemical stimulus (Additional file 1: Figure S2A). In fat body tissue, there were 2–10 times more transcripts upregulated in controls vs infected bees for primary metabolic processes, gene expression, ncRNA processing, regulation of apoptosis and mitochondrial membrane organization. The opposite pattern was only observed for lipid metabolic process (Additional file 1: Figure S2B).

### Effects of mixed *Nosema apis* and *Nosema ceranae* infection on fat body gene expression

Given that *N. ceranae* has achieved global prevalence and that co-infections in single bees occur naturally [14, 59], and also considering the chronic nature of microsporidian pathology, we conducted a second array study using *N. apis* and *N. ceranae* co-infected bees at a later time-point (14 days). While we are unable to directly compare impact of co-infection at this later time point to effects of *N. apis* infection at earlier time points in fat body tissue, together these studies provide insights into how microsporidian infection globally impacts worker metabolism.

PCR of midgut tissues confirmed that samples collected in 2010 were co-infected (Additional file 1: Figure S1B). We processed array data as before. After removing transcripts with expression levels below background, we included 12,596 transcripts in the analysis. A multivariate ANOVA identified 1,447 transcripts that were significantly, differentially regulated by *Nosema* co-infection (FDR < 0.0001; Additional file 2: Table S3).

GO analysis of these 1,447 transcripts (corresponding to 1,015 unique *Drosophila* orthologs) yielded overrepresented clusters involved in metabolism, cell organization and transport, development and immunity (Table 2). We determined the directional impact of infection on gene expression for transcripts included in significant GO categories. There were 3–17 times more transcripts that were upregulated in controls compared with those upregulated in infected bees within each significant GO category related to cellular organization and transport and metabolism. Only transcripts relating to immunity did not show strong trends in directional expression with equal numbers upregulated by both treatments (Additional file 1: Figure S2C).

**Table 2:** **Functional analysis of transcripts regulated by co-infection**

Functional category	GO term	GO biological process	***p***-value
**Metabolism**	GO:0042180	Cellular ketone metabolic process	2.42E-04
	GO:0006520	Cellular amino acid metabolic process	3.77E-04
	GO:0009152	Purine ribonucleotide biosynthetic process	1.71E-03
	GO:0044262	Cellular carbohydrate metabolic process	1.72E-02
	GO:0006629	Lipid metabolic process	2.31E-02
	GO:0042440	Pigment metabolic process	4.58E-02
	GO:0008152	Metabolic process	4.64E-02
**Cell organization and transport**	GO:0051234	Establishment of localization	3.56E-04
	GO:0006811	Ion transport	5.32E-04
	GO:0016192	Vesicle-mediated transport	2.10E-02
	GO:0033227	dsRNA transport	3.52E-02
**Other**	GO:0002376	Immune system process	1.99E-02
	GO:0046664	Dorsal closure, amnioserosa morphology change	2.28E-02

Transcripts significantly regulated by *Nosema* co-infection in worker fat body tissue were selected for functional analysis and overrepresented GO processes were identified.

### Comparisons with previous genomic studies

Dussaubat and colleagues (2012) identified 336 genes that were significantly regulated by *N. ceranae* in worker midgut tissue at 7 days pi (14 days old) [28]. Only 6 of these genes were also found to be regulated in midgut tissue in our study. This relatively small overlap is likely due to differences in inoculation time, inoculum dosage and species of *Nosema* used in these studies. However, examination of GO analyses revealed that both studies produced overrepresented biological clusters involved in midgut development, including genes related to neuronal and tracheal processes and midgut structure. In our study, genes involved in “regulation of neurogenesis” were affected while genes involved in “neuron differentiation”, “neuron development” and “axonogenesis” were regulated in Dussaubat et al. (2012). Also, in our study, genes related to “tube morphogenesis” were affected while genes involved in “open tracheal system development” and “morphogenesis of an epithelium” were regulated in Dussaubat et al. (2012).

Evans and colleagues identified canonical insect immune genes from the honey bee genome [57]. Of these, 166 genes were included on the array platform, which encompassed mediators of the major immune response pathways including Toll, IMD and JAK/Stat. No significant overlap (Hypergeometric Test; *p* > 0.05) was observed between any of our *Nosema* regulated transcript lists and the list of canonical honey bee immune genes (Table 3). We also compared our significantly regulated transcripts with a list of “acute” immune response transcripts regulated in the fat bodies of worker honey bees injected with saline, Sephadex beads or dead *E. coli* bacteria (evaluated at 6 hours pi) [58]. There was significant overlap with all three *Nosema*-regulated transcript lists (Table 3; Hypergeometric Test; *p* < 0.020 for midguts, *p* < 0.004 and *p* < 0.010 for fat bodies at 2 and 7 days pi and 14 days pi respectively).

**Table 3:** **Overlap of** ***Nosema*** **spp. regulated transcripts with canonical and acute immune response transcripts**

	Midgut	Fat body	Fat body	All significantly, differentially regulated transcripts or genes
	***N. apis*** (1 and 2 days pi)	***N. apis*** (2 and 7 days pi)	Co-infected (14 days pi)	
**Acute immune response transcripts [**[58]**]**	8*	14*	46*	**302**
**Canonical immune response genes [**[57]**]**	1	5	18	**166**
**All significantly, differentially regulated transcripts/genes (AM ids/GB ids)**	**150/97**	**278/225**	**1447/1108**	

*Indicates significantly more overlap than expected by chance (Hypergeometric Test; *p* < 0.02 at most).

Study results were compared with data from [58] and [57].

Finally, Ament and collaborators identified differentially expressed transcripts in the fat bodies of nurses versus foragers and workers fed on rich (pollen and honey) versus poor (sugar syrup) diets [43]. Significantly more overlap than expected by chance was observed between the diet transcript list and genes differentially regulated in the fat bodies of *N. apis* infected (2 and 7 days pi) and co-infected (14 days pi) workers (Hypergeometric Test; p < 0.009 and p < <0.0001 respectively; Table 4). Significantly more overlap than expected by chance was observed with the behavioral maturation (nurse vs forager) transcripts only with transcripts differentially expressed in the fat bodies of co-infected 14 day pi bees (Hypergeometric Test: p < <0.0001; Table 4).

**Table 4:** **Overlap of** ***Nosema*** **spp. regulated transcripts with behavioral state and diet lists**

	Fat body	Fat body	All significantly, differentially regulated transcripts
	***N. apis*** (2 and 7 days pi)	Co-infected (14 days pi)	
**Nurse vs forager**	52	425*	**2640**
**Rich vs poor diet**	87*	654*	**3351**
**All significantly, differentially regulated transcripts**	**278**	**1447**	

*Indicates significantly more overlap than expected by chance (Hypgeometric Test; *p* < 0.009 at most).

Study results were compared with data from [43].

Since overlap was strongest with diet and behavioral maturation transcript lists for co-infected samples (14 days pi), we next examined directional overlap among these sets of transcripts (Table 5). Notably, there was significantly more overlap than expected by chance across transcripts that were upregulated in controls and nurses, or bees fed on rich diets. There was also significantly more overlap than expected by chance across transcripts that were upregulated in *Nosema* co-infected bees and foragers, or bees fed on poor diets. Conversely, there was either no more overlap than expected by chance or significantly less overlap than expected by chance across controls and foragers, or bees fed on poor diets and across *Nosema* co*-*infected bees and nurses, or bees fed on rich diets. A Chi-Square test revealed that across studies, patterns of gene expression between controls and co-infected bees were more similar to expression patterns of bees fed on rich and poor diets respectively than to nurses and foragers (X^2^ = 93.72, *p* < 0.001).

**Table 5:** **Directional overlap of transcripts regulated by** ***Nosema*** **co-infection, worker behavioral state and diet**

Transcript list	Upregulated in co-infected bees (577 transcripts)	Representation factor, ( ***p*** -value)	Upregulated in controls (870 transcripts)	Representation factor, ( ***p*** -value)
**Upregulated in nurses (1204 transcripts)**	30	0.6, (*p* < 0.0003)	151	1.9, (*p* < <0.0001)
**Upregulated in foragers (1436 transcripts)**	149	2.4, (*p <* <0.0001)	95	1.0, (*p* < 0.4260)
**Upregulated in workers fed on a rich diet (1492 transcripts)**	13	0.2, (*p* < <0.0001)	342	3.5, (*p <* <0.0001)
**Upregulated in workers fed on a poor diet (1859 transcripts)**	265	3.3, (*p <* <0.0001)	34	0.3, (*p* < <0.0001)

Transcripts upregulated in controls or by *Nosema* co-infection (14 days pi) were overlapped with transcripts upregulated by worker behavioral state or diet treatments [43]. Significant overlap between transcript lists was identified using hypergeometric tests. A representation factor equal to one indicates no more overlap than expected by chance, a factor greater than one indicates more overlap than expected by chance, and a factor less than one indicates less overlap than expected by chance.

GO analysis of genes with the same directional overlap (203 unique *Drosophila* orthologues) for nurses and controls and foragers and infected bees yielded significant clusters (*p* < 0.05) related to metabolism and one cluster that was nearly significant related to immunity (p = 0.051) (Table 6). GO analysis of genes with the same directional overlap (417 unique *Drosophila* orthologues) for bees fed on a rich diet and controls and bees fed on a poor diet and infected bees yielded significant clusters (*p* < 0.05) related to metabolism, cellular organization and immunity (Table 7).

**Table 6:** **Functional analysis of transcripts regulated by** ***Nosema*** **co-infection and worker behavioral state**

Functional category	GO term	GO biological process	***p***-value
**Metabolism**	GO:0042180	Cellular ketone metabolic process	3.40E-04
	GO:0034754	Cellular hormone metabolic process	3.27E-03
	GO:0008152	Metabolic process	6.10E-03
	GO:0006189	‘De novo’ IMP biosynthetic process	7.81E-03
	GO:0006629	Lipid metabolic process	1.59E-02
	GO:0006066	Alcohol metabolic process	1.65E-02
	GO:0032787	Monocarboxylic acid metabolic process	2.34E-02
**Other**	GO:0006955	Immune response*	5.05E-02

*Not significant at p < 0.05.

Significantly regulated transcripts in the fat body tissue of co-infected workers (14 days pi) were overlapped with transcripts significantly regulated in the fat bodies of nurses and foragers [43]. Transcripts that were upregulated in controls and nurses or upregulated in infected bees and foragers were selected for functional analysis and overrepresented GO processes were identified.

**Table 7:** **Functional analysis of transcripts regulated by** ***Nosema*** **co-infection and worker diet**

Functional category	GO term	GO biological process	***p***-value
**Metabolism**	GO:0009152	Purine ribonucleotide biosynthetic process	3.46E-06
	GO:0006082	Organic acid metabolic process	7.30E-05
	GO:0008152	Metabolic process	1.22E-03
	GO:0009064	Glutamine family amino acid metabolic process	3.38E-03
	GO:0051186	Cofactor metabolic process	1.07E-02
	GO:0044262	Cellular carbohydrate metabolic process	1.71E-02
	GO:0032787	Monocarboxylic acid metabolic process	1.76E-02
	GO:0009167	Purine ribonucleoside monophosphate metabolic process	2.36E-02
	GO:0005996	Monosaccharide metabolic process	2.80E-02
	GO:0009056	Catabolic process	3.92E-02
	GO:0006119	Oxidative phosphorylation	4.46E-02
**Cellular organization**	GO:0051234	Establishment of localization	9.03E-03
	GO:0045184	Establishment of protein localization	2.14E-02
	GO:0016192	Vesicle-mediated transport	3.20E-02
**Immunity and other**	GO:0006955	Immune response	2.08E-02
	GO:0008063	Toll signaling pathway	2.72E-02
	GO:0050777	Negative regulation of immune response	3.08E-02
	GO:0050770	Regulation of axonogenesis	4.56E-02

Significantly regulated transcripts in the fat body tissue of co-infected workers (14 days pi) were overlapped with transcripts significantly regulated in the fat bodies of workers fed on rich and poor diets [43]. Transcripts that were upregulated in controls and workers fed on rich diets or upregulated in infected bees and workers fed on poor diets were selected for functional analysis and overrepresented GO processes were identified.

#### Candidate genes

Based on results from GO analyses and study comparisons, we selected candidate genes associated with metabolism, behavioral maturation and immunity that were significantly, differentially regulated in control versus co-infected workers at 14 days pi. Where data was available, we determined whether candidate genes were also upregulated in nurses versus foragers, and bees fed on rich versus poor diets [43]. Results of pairwise contrasts are summarized in Table 8. Selected genes were not necessarily significantly, differentially regulated in all three studies. Where differential expression was observed, a number of genes tracked directional expression expectations (e.g. upregulated across controls, nurses and/or bees fed on rich diets or were upregulated across co-infected bees, foragers and bees fed on poor diets). Other genes showed infection status by behavioral state (nurse vs. forager) or diet (rich vs. poor diet) interactions.

**Table 8:** **Expression patterns of select candidate genes regulating metabolism, development and immunity**

Function	Candidate genes	AM ID	Flybase ID	Control: Nosema	Nurse: Forager	Rich: Poor
**Insulin signaling, metabolism and behavioral maturation**	Insulin-like peptide 2	AM00001	FBgn0044050	Control	Nurseǂ	Richǂ
	PDK1	AM08362	FBgn0020386	Nosema	Forager	NR
	Akt1	AM08268	FBgn0010379	Control	NR	NR
	Foxo	AM06472, AM04383*	FBgn0038197	Nosema	NR	Poor
	Lipase-1	AM03164	FBgn0032264	Control	Nurse	Rich
	Lipid storage droplet 2	AM11728	FBgn0030608	Control	Foragerǂ	NRǂ
	Cpt1	AM11065	FBgn0027842	Control	Nurseǂ	Richǂ
	Usp	AM09226	FBgn0003964	Nosema	NR	NR
	Gigas	AM06808	FBgn0005198	Control	NR	NR
	Juvenile hormone epoxide hydrolase	AM03396	FBgn0010053	Control	Nurseǂ	Richǂ
	Juvenile hormone esterase	AM07915	FBgn0010052	Control	Nurseǂ	Richǂ
	Trehalose-6-phosphate synthase 1	AM05412	FBgn0027560	Nosema	Forager	Rich
	Venus kinase receptor	AM08479	No orthologue	Nosema	Forager	NR
**Immunity**	ECSIT	AM11800	FBgn0028436	Control	NR	Rich
	Pelle	AM08978	FBgn0010441	Control	Forager	Rich

*AM04383 was only significantly regulated in this study.

ǂData from qRT-PCR [43].

Directional expression of candidate genes was compared between this study (control:co-infected workers, 14 days pi) and expression in nurses versus foragers and workers fed on rich versus poor diets obtained from microarrays or quantitative real-time PCR [43]. Treatment names in the table indicate upregulation of a given transcript in the assigned treatment group relative to the other experimental group. NR indicates that no significant regulation was identified between groups.

Some of these candidate genes are associated with the immune system. For example, we found that *Nosema* co-infection regulated expression of members (*pelle, ECSIT)* of the Toll-like receptor signaling cascade, which is thought to mobilize insect immune effectors in response to challenge with fungi or gram + bacteria [56]. We also observed changes in *domeless* which is a central receptor in the JAK/Stat pathway [56].

Several of these genes are involved in nutritional and metabolic pathways. *Nosema* co-infection regulated expression of several members of the insulin signaling pathway (*Apis mellifera ilp-2; Akt1; Pdk1*) in addition to downstream transcription factors (*forkhead box, sub-group O*) [60]. As previously discussed, the insulin signaling pathway has been implicated in mediating worker behavioral maturation [50]. Co-infection also affected expression of *gigas,* which produces the Tsc2 protein in the *Drosophila* insulin signaling pathway. TSC2 links insulin signaling to the related TOR pathway which coordinates cell size and growth [61]. In honey bees, initial studies indicate that changes in TOR signaling may also affect behavioral maturation [50]. We also saw significant regulation of *venus kinase receptor* (*vkr*) which belongs to a recently discovered, nutrient-sensitive family of receptor tyrosine kinase proteins found in some invertebrates. Expression of *vkr* is correlated with development in male honey bees and reproduction in other model invertebrates [62]. As previously discussed, abdominal lipid content is an important physiological factor that contributes to worker behavioral maturation [42, 44]. Co-infection regulated several genes involved in lipid metabolism (*lipase-1, lipid storage droplet 2; carnitine O-palmitoyl transferase 1*) that have been previously correlated with worker behavioral state and/or diet [43]*.* Interestingly, *lsd-2* expression is also modulated by acute infections [58].

Co-infection upregulated expression of *ultraspiracle* (*usp*), a transcription factor that responds to JH titers and that may act in concert with other factors to regulate behavioral maturation. For example, *usp* knockdown slows onset of foraging behavior [63]. Co-infection also modified expression of *juvenile hormone esterase* (*Jhe)* which likely breaks down JH in worker honey bees [64] in addition to expression of *juvenile hormone expoxide hydrolase* (*Jheh*). Previous studies suggest the *Jheh* does not break down JH in honey bees as in other insects, but is responsive to worker behavioral state and diet [43, 65].

### Validation of gene expression results using quantitative real-time PCR

Given that the number of canonical immune response genes regulated by *Nosema* infection was low, we used qRT-PCR to examine expression levels of five candidate genes with immune (*abaecin, hymenoptaecin, defensin-1*), nutritional and/or developmental functions (*vitellogenin, hexamerin 70b*) across treatment groups for RNA extracted from fat body tissue at 7 and 14 days. Gene expression was standardized to *actin*. Results indicated that all genes at 7 days pi followed a general trend for upregulation in controls relative to *N. apis* treated bees, however none of these trends were significant (Mann–Whitney U Test, p > 0.05; Additional file 1: Figure S3A). Results at 14 days pi (co-infected bees) revealed a more heterogeneous pattern of expression though no differences in expression were significant across treatment groups (Mann–Whitney U Test, p > 0.05; Additional file 1: Figure S3B). We also verified that no corresponding transcripts for these genes were significantly differentially expressed on the microarrays (Additional file 2: Table S4).

## Discussion

Gene expression analysis suggests that infection with *Nosema apis* has immediate, deleterious consequences for midgut structure and function. Resulting tissue damage and subsequent siphoning of host resources may significantly and negatively impact metabolic and nutritional pathways in worker fat body tissue. Ultimately, for chronologically advanced co-infections, metabolic and nutritional changes may affect expression of genes linked to worker behavioral maturation, predisposing infected individuals to foraging tasks. Altered expression of these genes may also simultaneously drive changes in worker immune function, though it remains to be determined if immune pathways are altered by nutritional deprivation, behavioral maturation, increasing amounts of tissue damage and/or directly by infection.

Importantly, when synthesizing results across studies, our findings must be interpreted with caution due to different time points and species of infection used in these three microarray analyses. In addition, by caging study subjects in these experiments we strongly controlled worker environment and standardized food access. This reduced variability in gene expression, facilitating identification of *Nosema-*mediated changes. However, as individual worker nutritional and behavioral status are closely linked to colony signals and resources, future studies examining gene expression in workers housed in colonies are needed to validate findings from this study. Finally, additional studies employing pure *N. apis* and *N. ceranae* infections may also underscore similarities and differences in pathology and virulence of each parasite.

### Effects of *Nosema apis* infection on worker midgut development

Our results suggest that *N. apis* infection may impair midgut development at early time points, since genes involved in molecular processes likely associated with repairing tissue damage were significantly regulated. Interestingly, along with genes involved in neurogenesis and tube morphogenesis, we saw significant regulation of *headcase*[66] and *slit*[67] which interact with other factors to respectively mediate tracheal branching and directional growth in fruit flies. Since the trachea supply tissues with oxygen, these changes may be due to tissue repair mechanisms and/or changing respiratory demands of midgut cells as they begin to support parasite propagation. Indeed, previous studies by Dussabaut and colleagues (2012) examined the impact of *N. ceranae* infection in worker midgut tissue and found that similar GO categories, including tracheal development, were affected at 7 days post-infection (but 14 days of age). *Sli* was also regulated in this study [28] and incorporated in a larger network of genes involved in tissue regeneration (Wnt signaling pathway).

### Effects of *Nosema* infection on worker immunity

Previous studies suggest that *Nosema* infection alters expression of canonical immune genes in abdominal tissues, though this response is modulated by *Nosema* species, spore load, infection period/age of bee, and genotype of the bee [32, 33, 68]. Similarly, studies examining interactions between bumble bees and their intestinal, trypanosomal parasites have shown that host expression of immune genes is modulated by infection period [69]. Interestingly, in these experiments, genes found responding to acute immunostimulation in a previous study [58], but not canonical immune genes [57] as a group were significantly regulated across all infection types, tissues and timepoints, suggesting that non-canonical immune genes may be important in moderating local and systemic defense responses to *Nosema*. However, in co-infected workers, we did note changes in select members (e.g. *pelle, ECSIT*) of the Toll signaling pathway which is thought to defend insects against fungi [56]. Interestingly, members of the Toll signaling pathway were differentially regulated in drones from selected, *N. ceranae* tolerant, Danish lineages and unselected German lineages, suggesting that the Toll signaling pathway may play an active role in honey bee defense against this pathogen [68]. Given the temporally dynamic nature of immune responses, the high degree of cross-talk amongst pathways and the likely role of non-canonical immune genes, future immune studies may track broader suites of candidate genes over longer time courses. Furthermore, local constitutive immune responses, not captured in transcriptional studies, may play a role in defense as suggested by [28].

### Effects of *Nosema* infection on worker fat body metabolism; links to worker nutrition and behavioral maturation

Consistent with previous studies indicating that infection is energetically costly, we found that *Nosema* infection broadly altered expression of genes involved in metabolic processes in worker fat body tissue. Metabolic changes were evidenced at earlier (2 and 7 day) timepoints, where *N. apis* infection impacted lipid metabolism and mitochondrial membrane organization. At later time points in co-infected bees, expression of transcripts related to carbohydrate, protein and lipid metabolism was reduced relative to controls. Previous studies demonstrate that the fat body can direct nutrient-sensitive transitions in worker behavioral maturation. Thus infection-mediated changes in this tissue’s metabolism may in part drive documented precocious foraging behavior. Indeed, comparative analyses validated both metabolic and maturational changes in co-infected worker gene expression profiles. Also, genes previously implicated in both worker nutrition and maturation were regulated in this study. Candidates from the insulin signaling pathway that were regulated in this study (*Apis mellifera ilp-2; Akt1; Pdk1; foxo, gigas*) may be of particular interest for future studies investigating molecular mechanisms behind symptoms of *Nosema* infection. Current theory suggests that insulin signaling in worker honey bees performs both a conserved role in regulating individuals’ nutritional balances and an evolutionarily co-opted role in division of labor by contributing to worker task-orientation [40]. Thus, at the molecular level, changes in the insulin signaling pathway may reflect energetic costs of *Nosema* infection and reflexively promote changes within the same pathway or linked networks that stimulate precocious foraging.

### Modeling the direct and indirect costs of *Nosema* infection on workers

**Figure 2:**
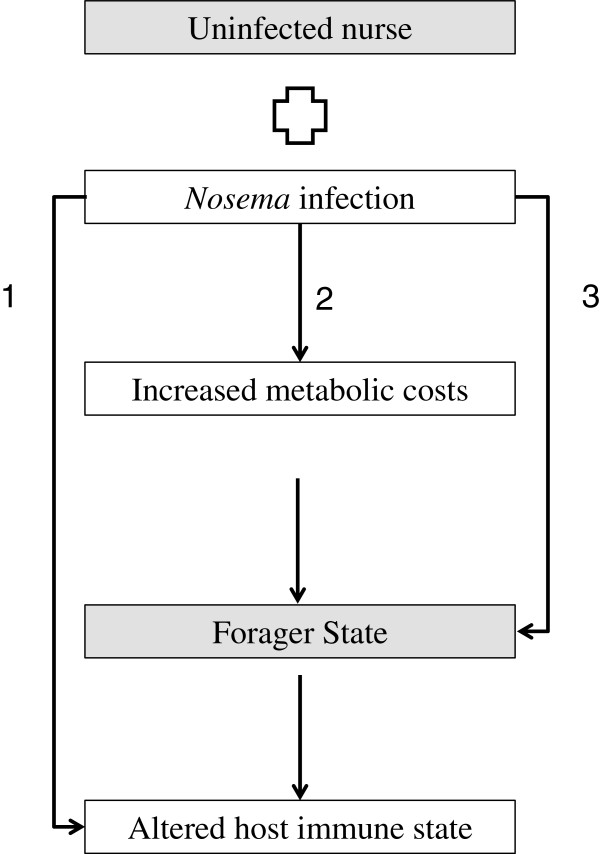
**Proposed model for how** ***Nosema*** **infection impacts worker metabolism, hormonal signaling and immunity.** 1) Infection with *Nosema* may directly trigger expression of immune genes independent from worker behavioral state, 2) Infection with *Nosema* may damage nurse midgut tissue and retard midgut development, resulting in reduced nutrient uptake. Poor nutrition may be compounded by energetic costs exerted by *Nosema* parasites. Increased worker metabolic costs may alter worker genes regulating hormones and stress response to dietary restriction. Changes in expression of these genes may accelerate worker behavioral maturation, driving them towards a foraging state, which in turn alters worker immune state. 3) *Nosema* parasites may directly alter expression of genes regulating behavioral maturation, driving workers towards a foraging state with associated changes in immune function. These hypotheses are not mutually exclusive.

Based on these findings, we suggest three possible models (Figure 2) for how *Nosema* affects worker physiology, immunity and behavioral maturation. First, *Nosema* infection may directly regulate expression of immune genes. Second, *Nosema* may ‘starve’ its hosts through destruction of midgut tissue and/or by appropriating host resources, resulting in accelerated behavioral maturation and associated changes in worker hormones, metabolism and immunity. Third, *Nosema* may directly impact expression of genes regulating behavioral maturation leading to associated changes in metabolism and immunity. These pathology scenarios are not mutually exclusive and may operate in concert to produce symptoms of infection. To better illuminate the relative contributions of restricted nutrition/impaired metabolism in infected workers versus direct manipulation of gene expression in driving precocious foraging in infected workers, we determined whether gene expression profiles of co-infected workers were more similar to that of bees fed on poor diets or foragers [43]. Results from a Chi-Square test found that infected workers were more similar to bees fed on a poor diet than foragers. Workers were caged in this study and the diet study, but not the behavior study, which may have contributed to overall similarity between this study and the diet study. However, as demonstrated by directional overlap analyses (see Table 5), treatment (infection status or diet) is a primary driver of similarity between studies. Taken together, these analyses, highlight the second model as an important pathway for *Nosema* disease etiology. Additionally, GO analyses of overlapping transcripts with the same directional expression between this study (14 days pi, co-infected workers) and diet and maturation gene lists both produced significant or near-significant immune categories (Tables 6 and 7). These findings suggest a complex relationship between worker immunity, behavioral state and hormonal and metabolic profiles. Indeed, studies in insects and other animals point to the importance of nutrition in optimizing immunity [70]. The impoverished nutritional/metabolic status of infected workers may compromise their immune defenses though additional studies employing secondary pathogen challenges are needed to test this hypothesis.

### Common responses to diverse stressors in honey bees

Worker genomic responses to *Nosema* infection may also represent common, chronic stress response pathways in honey bees. In mammals, acute stress impels catabolism of energy stores to fuel fight-or-flight responses but chronic stress can lead to dysregulation of the molecular pathways sustaining the stress response [71]. Negative, interlinked consequences of prolonged stress, inflammation and metabolic dysfunction have been well-documented in mammalian models [72, 73]. Though additional work is required to characterize responses to immediate and protracted stress in honey bees, several factors (e.g. biogenic amines, neuropeptides, and hormones regulating energy metabolism) have been identified as candidate mediators. In addition, vitellogenin, JH and the insulin signaling pathway, serving as metabolic regulators of stress responses and/or molecular buffers against stress, in addition to other regulators of division of labor, have also been listed as potential contributing agents [71]. Thus, stress, distinct from energetic costs of infection, may simultaneously function as a response to and catalytic driver of the metabolic symptoms of *Nosema* infection and subsequently lead to changes in worker immunity and task-related behaviors. Indeed, studies examining gene expression in *N. ceranae* infected midgut tissue point to the likely involvement of a canonical stress hormone, CRH-BP (corticotrophin-releasing hormone binding protein) [28].

Precocious foraging as a general, behavioral stress response has been previously noted in honey bees. For example, immune challenge with non-replicating pathogens, such as injection with LPS, induces forager-like gene expression, physiology and behavior in treated workers compared with Ringer-injected controls [74]. Workers subjected to wounding via injection and CO_2_-treated workers forage sooner than non-injected or untreated controls, suggesting that diverse stressors can be powerful instigators of behavioral maturation [35, 48, 49]. Parasitization with *Varroa* mites also results in accelerated behavioral maturation [75] and alters expression of members of the insulin and TOR pathways in addition to other molecular markers associated with precocious foraging [76]. *Varroa* mite (and associated viral) parasitization depresses worker expression of genes related to metabolism, especially protein metabolism [76] and reduces protein content in adult hemolymph, including Vg [77]. However, additional studies indicate that *Varroa* and associated viral infections may have complex effects on worker protein content and other nutritional markers in workers [78]. Also, not all parasites universally induce precocious foraging in honey bee hosts [75]. Thus, future studies examining common physiological and behavioral outcomes in honey bees to disparate stressors will enhance our understanding of the stress response in this insect model [71].

Overall, precocious foraging, as a byproduct of energetic stress levied by *Nosema* infection (previously suggested by [23, 25]) and/or a byproduct of the worker stress response to infection, carries costs for individuals and colonies. As previously outlined [11], because precocious foraging results in premature death, and because *Nosema* infected workers may have greater levels of extrinsic mortality due to other aberrant behavioral or physiological symptoms of infection, *Nosema* infected colonies face additional energetic costs of rearing replacement workers, who may also forage prematurely. This may result in weaker colony populations, poor food stores [12] and potentially colony death [79]. However, despite costs associated with premature foraging, colonies may also benefit from reduced within-hive pathogen transmission as suggested by [37]. Further studies integrating changes in individual physiology and behavior and colony regulation of division of labor will enhance our understanding of how social structures allocate resources in response to energetically costly infections.

## Conclusions

Our studies demonstrate the complexity of examining the impacts of chronic disease and parasitization on individuals and social groups. The direct and indirect effects of disease or parasitism can be difficult to disentangle. However, by combining timecourses, tissue-specific analyses and genomic tools it is possible to begin to elucidate the molecular mechanisms that mediate disease symptoms, at the level of the individual and ultimately the social group. In the case of *Nosema*, the disruption of gut integrity and siphoning of host energy may trigger downstream changes in key, interconnected metabolic and hormonal pathways, resulting in changes in host maturation and, thus, ultimately, productivity and longevity. Through comparative analyses we identified molecular pathways underpinning this response, including the insulin signaling pathway. We also discuss the interlinked roles of energetic stress induced by *Nosema* parasitism and stress as a host response to infection. Future studies are needed to elucidate the relative contribution of each of these two types of stress to catalyzing disease symptoms. Changes in individual worker maturation as a consequence of infection likely affect both disease transmission dynamics and colony age-structure and organization, thereby impacting colony productivity and health.

## Methods

### Worker infection

Honey bee colonies headed by Kona Italian queens (Kona Queen Hawaii, Inc, Captain Cook, HI) were maintained in the Weslaco, TX apiary according to standard beekeeping practices. In 2008, frames of late-stage brood were placed in an incubator at 33°C for 24 hours to obtain newly emerged workers. Workers were placed in cages (12 x 17 x 9 cm) with 100 bees per cage. Fifty workers were randomly selected from each cage, restrained, and fed 5 ul of 50% sugar solution with or without 50,000 *Nosema apis* spores. Workers were marked and returned to their cage. Cages were maintained in an incubator at 33°C, 50% RH and kept in the dark. Bees were fed MegaBee (MegaBee, Yuma, AZ) as a pollen-substitute and protein source and 50% sugar water ad libitum. Megabee- and pollen-fed caged bees show similar physiological development [80]. Also, rearing *Nosema* infected bees in cages with supplementary diets has been done previously [28, 32, 36] and physiological impacts of *Nosema* infection have been demonstrated in cage and field studies [23–25, 32, 36, 52]. Bees were collected on dry ice at 1, 2 and 7 days pi (post-infection) and stored at -80°C. *Nosema* spores were amplified in caged bees prior to the experiment at the USDA laboratory in Weslaco, TX. *N. apis* spores originated from a long standing culture maintained at Kentucky State University.

In 2010, *N. apis* spores were obtained as before and *N. ceranae* spores were isolated from a single bee collected in a heavily infected commercial apiary (LA, USA). Spores of both species were amplified in cages and fed to newly emerged workers as before. This time, however, workers were fed 25,000 spores of *N. apis* and 25,000 spores of *N. ceranae* and were collected on dry ice after 14 days. All samples were stored at -80°C.

Samples from both years were shipped to Penn State University on dry ice, and RNA from the migduts of samples collected in 2008 was converted to cDNA and tested for *Nosema* infection using *N. apis* and *N. ceranae* specific primers (Additional file 2: Table S5) using conventional PCR and reaction conditions as in [81, 82]. In 2010, midguts were homogenized in RLT/BME buffer (Allprep DNA/RNA/Protein Mini Kit, Qiagen, Valencia, CA) and an aliquot was taken for DNA extraction. Briefly, 90 ul of each homogenate was incubated overnight with 125 ul of a CTAB buffer (100 mM Tris–HCl (pH 8.0), 20 mM EDTA (pH8.0), 1.4 M NaCl, 2% Cetyltrimethylammonium bromide, 0.2% 2-mercaptoethanol) at 56°C with 20 ul of proteinase K from the Allprep kit. (CTAB protocol courtesy of Dr. Judy Chen, USDA-ARS, Beltsville. CTAB buffer breaks down microsporidia spore walls). In the morning, the sample was added to Allprep columns and DNA was extracted. DNA was checked for *Nosema* spp. infection using conditions in [81, 82].

### Dissection, RNA extraction and array hybridization

We thawed individual bees on ice-chilled, sterile plates under cold RNAlater (Qiagen, Valencia, CA) and dissected midguts from bees collected 1 and 2 days pi (post-infection) in 2008. We dissected fat bodies (eviscerated abdomens with digestive and reproductive organs and venom sac removed) at 2 and 7 days pi from bees collected in 2008 and at 14 days pi from bees collected in 2010. We pooled tissues from 5 bees per biological replicate (n = 4 per tissue and timepoint).

In 2008, RNA was extracted from midgut and fat body samples with Trizol (Invitrogen, Carlsbad, CA) and amplified with the Ambion MessageAmp II aRNA Amplification Kit (AM1751, Life Technologies, Grand Island, NY). Each sample was labeled independently with Cy3 or Cy5 using the ULS aRNA fluorescent labeling kit (EA-006, Kreatech, Amsterdam, Netherlands) and hybridized with the Maui Hybridization System (BioMicro Systems, Salt Lake City, UT) to whole genome, oligonucleotide microarrays [83] obtained from the Keck Center for Comparative and Functional Genomics (University of Illinois, Urbana-Champaign, USA). Arrays were scanned with the Axon Genepix 4000B scanner (Molecular Devices, Sunnyvale, CA) and viewed with GENEPIX software (Agilent Technologies, Santa Clara, CA). In 2010, RNA was extracted from fat body samples with the RNeasy Kit (Qiagen, Valencia, CA) and amplified, labeled and hybridized as before. For each tissue and timepoint, we used a loop design incorporating dye swaps that resulted in 16 arrays for midgut tissue, 16 arrays for fat body tissue in 2008 and 8 arrays for fat body tissue in 2010. Thus each array experiment incorporated 4 biological replicates per treatment and 2 technical replicates per sample.

### Microarray data analysis

The 2008 midguts, 2008 fat bodies, and 2010 fat bodies were analyzed as three separate studies. For all analyses, we removed spots with an intensity less than 100 (the intensity level of the array background) in addition to transcripts with less than 7 observations from each analysis. We log-transformed and normalized expression data using a mixed-model ANOVA in SAS (proc MIXED, Cary, NC):

where Y is expression, dye and block are a fixed effects, and array, array*dye and array*block are random effects. Genes with significant expression differences between groups were detected by using a mixed-model ANOVA.

We used the following model for midguts and fat bodies collected in 2008:

where Y represents the residual from the previous model. Treatment, age, treatment*age, spot and dye are fixed effects and array is a random effect.

We used the following model for fat bodies collected in 2010:

where Y represents the residual from the previous model. Treatment, spot and dye are fixed effects and array is a random effect.

*p*-values were corrected for multiple testing using a false discovery rate of FDR < 0.001 for midgut and fat body tissue collected in 2008 (proc MULTTEST, SAS). *p*-values for fat body samples collected in 2010 were corrected for multiple testing using an FDR < 0.0001. Because a greater number of transcripts were affected in the latter study, we chose a more stringent FDR value, allowing for a more targeted analyses of core genes that were regulated by *Nosema* co-infection. The array datasets supporting the results of this article are available at the ArrayExpress repository (*N. apis* infection in worker midgut tissue: E-MEXP-3891, *N. apis* infection and worker fat body tissue: E-MEXP-3892, Co-infection and worker fat body tissue: E-MEXP-3889).

We conducted principal components analysis (JMP 9.0.2, SAS, Cary, NC) to determine relative impact of treatment, age and age x treatment interactions on gene expression. Genes with a significant treatment effect only were selected for subsequent analyses. Gene ontology (GO) analysis was performed using the Functional Annotation Tool from DAVID (version 6.7) [84, 85] with a significance cutoff of *p* <0.05. For all GO analyses, array transcripts were matched to Flybase orthologs and the entire array transcript list with matches to Flybase orthologs was used as a background list (7,186 unique Flybase genes). Note that the oligos on the arrays were annotated in 2007 [86]. Candidate gene annotations presented in Table 8 were manually annotated by blasting oligo nucleotide probe sequences from the arrays, and then selecting protein sequence of the best honey bee gene match and blasting these protein sequences against the translated *Drosophila melanogaster* genome. Reciprocal blasts were performed on some genes to check accuracy of identification.

### Comparisons with previous studies

We compared transcripts/genes significantly regulated by infection status with transcripts/gene lists from five other studies, described below.

1. ***Nosema ceranae*genes** (midgut tissue) [28]: Genes significantly and differentially regulated in control workers vs workers infected for 7 days with *N. ceranae.*
2. **Canonical immune genes**[57]: Canonical immune genes identified during annotation of the honey bee genome.
3. **Acute immune response transcripts** (fat body tissue) [58]: Transcripts significantly and differentially regulated by immune challenge (bacteria-, Sephadex bead-, or saline-injection) in worker bees
4. **Nurses versus foragers** (fat body tissue) [43]: Significantly, differentially regulated transcripts between nurses and foragers.
5. **Workers fed on rich versus poor diets** (fat body tissue) [43]: Significantly, differentially regulated transcripts between workers fed on rich (honey and pollen) and poor diets (sugar syrup).

For these study comparisons, note that the transcripts on the microarrays are designated with “AM” (*Apis mellifera)* numbers [86]. These transcripts correspond to annotated genes from the honey bee genome, which are annotated with “GB” (GBrowse) numbers [87]. For comparisons with gene lists generated from the honey bee arrays (#3-5), it was possible to directly compare gene lists based on transcript AM numbers. For comparisons with other studies (#1-2) which investigated whole genome data but did not use microarrays or used tiling arrays, it was necessary to use GB annotations for microarray transcripts rather than AM notations. We identified overlap between studies using an online Venn diagram service [88] and tested for significance using a hypergeometric test [89]. Background overlap of between studies was assumed to be either the entire list of AM numbers (studies #3-5) or GB numbers on the arrays (#1-2). Select GO analyses based on study overlap were performed in DAVID as above.

### Validation of gene expression using quantitative real-time PCR

We examined gene expression levels of three antimicrobial peptides involved in immune function (*abaecin*, *defensin-1*, *hymenoptaecin*) [57] and expression of two additional genes involved in worker maturation and/or nutrition (*vitellogenin*[45], *hexamerin 70b*[90]). We used the total RNA extracted from worker fat bodies for the microarray analysis and compared gene expression between bees with *Nosema apis* infections and controls at 7 days pi, and gene expression between bees with mixed *Nosema* infections and controls at 14 days pi. cDNA was synthesized using SuperScript II First-Strand Synthesis System for RT-PCR (Invitrogen-Life Technologies, Carlsbad, CA, USA) and oligo-dT primers according to the manufacturer’s protocol. The cDNA was then diluted 10(x) with double distilled autoclaved water. Amplification was performed in a 10 μl reaction mixture containing reaction mix of 0.5 U of GoTaqR Flexi DNA polymerase (Promega Co., Madison, WI) with the colorless 5× GoTaqR Flexi buffer, 0.38 mM dNTP mix, 5.0 mM MgCl_2_, 0.35 μM of each primer, 0.33 μl of a 1/1000 stock dilution of SYBR-Green (Invitrogen Corp.), and 1 μl of cDNA. Reactions were loaded on the CFX96™ Real-Time PCR Detection System (BioRad Ltd, Hercules, CA) and run for 40 cycles at 95°C (5 s) and 60°C (30 s) after initial denaturing at 95°C for 3 minutes. Fluorescence was measured at the end of the annealing stage of every cycle. A final extension of 72°C (2 mins) followed by a melting curve analysis was added to the program to ensure that true product was being amplified. Negative control reactions were included in each run and contained all reaction components except the template. This helped discern any primer dimers that may have amplified. Individual samples were standardized against *actin* expression levels. Primers used for these genes were previously published [78] and can be found in Additional file 2: Table S6. Relative fold expression of candidate genes between treatment groups was calculated and differences in gene expression were tested for significance with Mann Whitney U Tests in JMP 10 (Cary, NC).

## Electronic supplementary material

Additional file 1: Figure S1-S3: This file includes supplementary figures documenting PCR results, directional regulation of transcripts within significant GO categories and qRT-PCR results. (DOCX 478 KB)

Additional file 2: Tables S1-S6: Supplementary Tables 1-6 include lists of significantly regulated transcripts identified in this study as well as transcript annotation. These tables also include data about primers used for study validation and results of qRT-PCR analyses. (XLSX 569 KB)

## Abbreviations

JH: Juvenile hormone
vg: Vitellogenin
pi: Post-infection
GO: Gene ontology
IMD: Immunodeficiency
JAK/Stat: Janus kinase/signal transducer and activator of transcription
ECSIT: Evolutionarily conserved signaling intermediate in toll pathway
AmIlp-2: Apis mellifera insulin-like peptide 2
Pdk1: 3-phosphoinositide-dependent protein kinase 1
foxo: Forkhead box protein O
Akt: Serine/threonine protein kinase
Jhe: Juvenile hormone esterase
Jheh: Juvenile hormone epoxide hydrolase
Usp: Ultraspiracle
NF-kB: Nuclear factor kappa B
JNK: c-Jun amino-terminal kinase
AMP: Antimicrobial peptide
Cpt1: Carnitine O-palmitoyl transferase 1
lsd-2: Lipid storage droplet 2
TOR: Target of rapamycin
VKR: Venus kinase receptor
NR: Not regulated
CRH-BP: Corticotrophin-releasing hormone binding protein.
