# Supplementary material for: Chronic parasitization by Nosema microsporidia causes global expression changes in core nutritional, metabolic and behavioral pathways in honey bee workers (Apis mellifera)
Source: BMC Genomics. 2013 Nov 18;14(1):799. doi: 10.1186/1471-2164-14-799 (PMC4046765; doi:10.1186/1471-2164-14-799)
Supplement: Supplementary file 1 — Additional file 1: Figure S1-S3: This file includes supplementary figures documenting PCR results, directional regulation of transcripts within significant GO categories and qRT-PCR results. (DOCX 478 KB) [file 12864_2013_5525_MOESM1_ESM.docx]

**Supplementary Figure 1: Verification of sample infection status.** Select samples were tested for infection with *N. apis* (16s) and *N. ceranae* (SWP32) specific primers. **A)** **PCR of samples for *N. apis* arrays (2008).** Amplification of *Nosema apis* 16s primers was observed for a subset of tested samples infected with *Nosema* (indicated by ‘Na’ in sample label) across timepoints (‘7d’ = 7days, ‘48’ = 2 days). A tested subset of control samples did not show *Nosema apis* infection. No samples showed evidence of *Nosema ceranae* (SWP32 primers did not amplify for any samples). **B)** **PCR of samples for co-infection arrays (2010).** Primers for *Nosema apis* (16s) and *Nosema ceranae* (SWP32) amplified for all infected samples (indicated by ‘NaNc’ in sample label). A tested subset of control samples (indicated by ‘C’ in sample label) did not show evidence of infection with either *Nosema* species.

**Supplementary Figure 2: Directional expression of transcripts in significant GO categories.** We tallied the number of individual transcripts upregulated by control or *Nosema* spp. infection within each significant GO category. **A)** **Midgut tissue at 1 and 2 days pi*:*** *N. apis* infection increased expression of transcripts involved in ‘regulation of neurogenesis’, ‘tube morphogenesis’ and ‘multicellular organismal process’ but decreased expression of transcripts involved in ‘sensory perception of chemical stimulus.’ **B)** **Fat body tissue at 1 and 2 days pi:** *N. apis* infection reduced expression of transcripts involved in all significant GO categories, except for those related to ‘lipid processes.’ **C)** **Fat body tissue at 14 days post-infection:** *Nosema* co-infection generally suppressed expression of transcripts with cellular transport and metabolism functions. However, approximately equal numbers of transcripts involved in ‘immune processes’ were upregulated by both treatments.


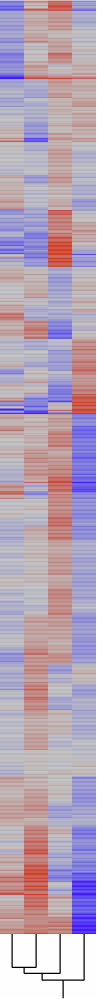


**Controls, 1 day pi**

**Controls, 2 days pi**

***Nosema*, 1 day pi**

***Nosema*, 2 days pi**


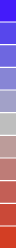

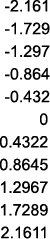


**Fig. 1a: Midguts**

**Supplementary Figure 3: Quantitative real-time PCR validation of expression patterns of immune, developmental and nutritional genes.** Expression levels of three antimicrobial peptide genes (*abaecin*, *defensin*, *hymenoptaecin*), *hexamerin* and *vitellogenin* (relative to *actin*) from array samples were analysed using quantitative real-time PCR. Mean expression levels for each treatment group were normalized to expression in the control treatment group. Expression was analysed in **A) Fat body tissue from 7-day old controls and workers with *N. apis* infection (n=4, 2008 samples)**, and **B) Fat body tissue from 14-day old controls and workers with *N. apis* and *ceranae* co-infections (n=4, 2010 samples)**. No significant differences in expression levels across the treatment groups were detected with Mann-Whitney U Tests (*p*>0.05).

**Supplementary Figure 1A:**

**
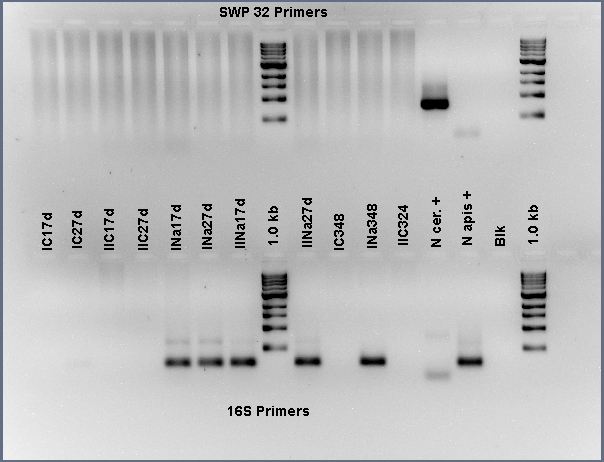
**

**Supplementary Figure 1B:**


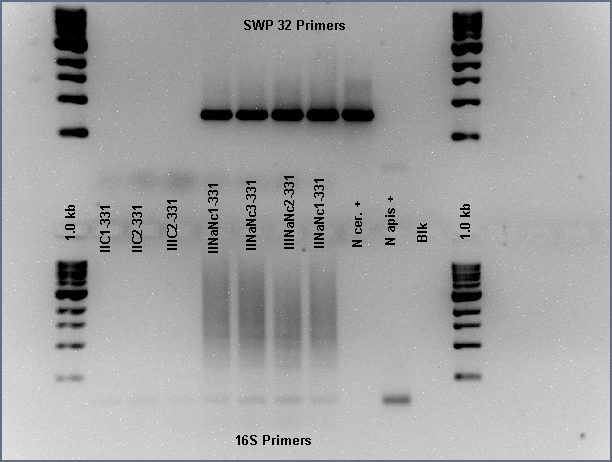


**Supplementary Figure 2A:**

**Supplementary Figure 2B:**

**Supplementary Figure 2C:**

**Supplementary Figure 3A:**

**Supplementary Figure 3B:**

**Fig. 1a: Midguts**

**Fig. 1a: Midguts**

**Fig. 1a: Midguts**
